# Loss of Biventricular Capture After CRT-D Implantation: Device Malfunction or Something Else?

**DOI:** 10.1016/j.jaccas.2026.110182

**Published:** 2026-09-02

**Authors:** Paul Weber, Dhruva Govil, Dipak Shah

**Affiliations:** a Department of Cardiology, Henry Ford Health Providence, Southfield, Michigan, USA; b Department of Internal Medicine, Henry Ford Health Providence, Southfield, Michigan, USA

**Keywords:** cardiac resynchronization therapy, chronic heart failure, electrocardiogram

## Abstract

**Case Summary:**

A 75-year-old woman with nonischemic cardiomyopathy and left bundle branch block underwent cardiac resynchronization therapy defibrillator implantation. Postimplant electrocardiography demonstrated successful biventricular pacing with QRS narrowing to 110 ms from a preimplant baseline of 169 ms. Three consecutive beats with an abruptly different QRS morphology raised concern for intermittent loss of biventricular pacing or device malfunction.

**Take-Home Message:**

Dynamic atrioventricular-delay optimization algorithms such as SyncAV can produce transient changes in QRS morphology that may mimic loss of biventricular pacing but represent expected device behavior and recognition of this pattern prevents unnecessary device evaluation.

## Graphical Abstract

**Figure:**
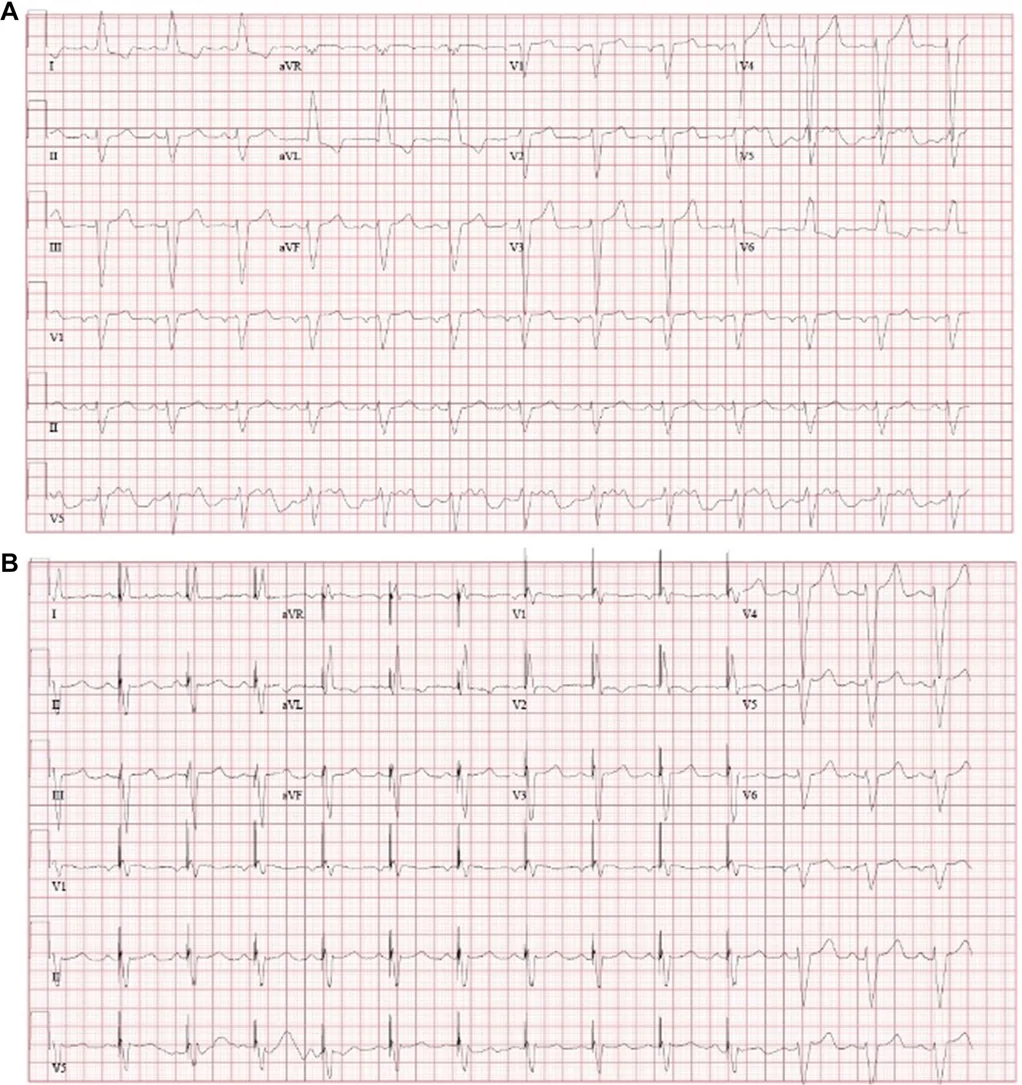

## Clinical Presentation

A 75-year-old woman with nonischemic cardiomyopathy, NYHA functional class II heart failure, left ventricular (LV) ejection fraction <35%, and left bundle branch block (LBBB) despite guideline-directed medical therapy was referred for cardiac resynchronization therapy (CRT)-defibrillator implantation. The preimplant electrocardiogram (ECG) (Figure 1A) shows native LBBB, QRS 169 ms.

**Figure 1:**
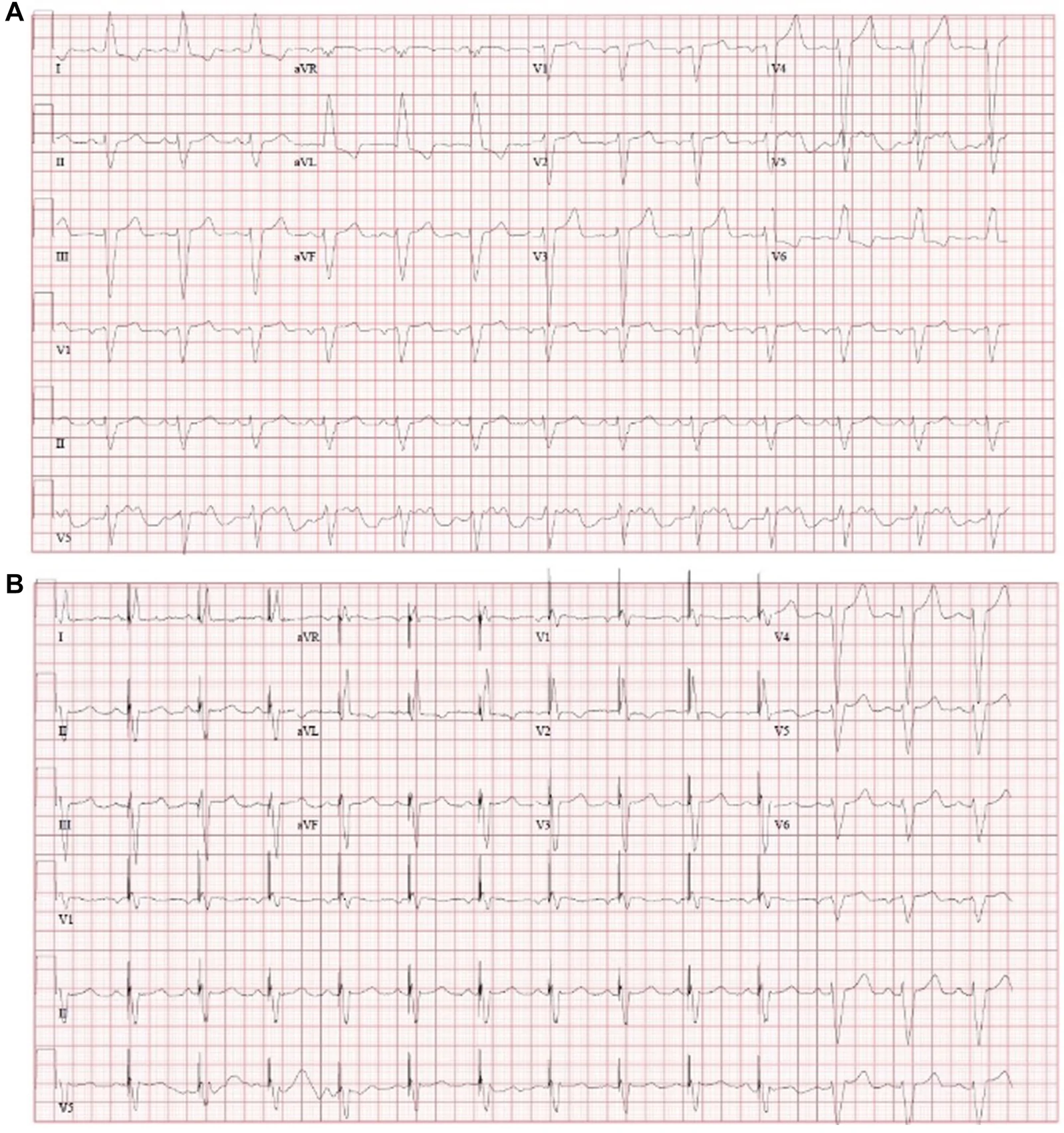
Electrocardiogram Pre- and Post-Implant (A) Pre-implant 12-lead electrocardiogram demonstrating native left bundle branch block morphology with a QRS duration of 169 ms. (B) Post-implant 12-lead electrocardiogram demonstrating A-sensed V-paced rhythm with SyncAV-mediated triple fusion pacing. QRS duration narrows to 110 ms (35% reduction from baseline). The final 3 complexes exhibit a wider, different QRS morphology.

An Abbott CRT-defibrillator system (Gallant HF) was implanted with a quadripolar LV lead in the anterolateral coronary sinus branch (Q-to-LV sensing interval 105 ms). The device was programmed DDD at 60 to 130 beats/min. The postimplant ECG is shown in Figure 1B.

Based on the ECG in Figure 1B, what is the most likely explanation for the 3 beats with abruptly different QRS morphology?

A. Loss of left ventricular capture with unopposed right ventricular apical pacing
B. Automatic mode switch to a nontracking ventricular pacing mode triggered by atrial tachyarrhythmia detection
C. Loss of right ventricular capture with unopposed LV pacing
D. SyncAV algorithm–mediated atrioventricular (AV) delay extension for periodic intrinsic conduction reassessment

Correct Answer: D. SyncAV algorithm–mediated AV delay extension for periodic intrinsic conduction reassessment.

Figure 1A shows native LBBB (QRS 169 ms). Figure 1B demonstrates biventricular pacing with QRS narrowing to 110 ms (35% reduction) via triple fusion. During the final 3 beats, the QRS widens and assumes a different morphology before returning to the narrow fusion pattern. Interrogation confirmed no threshold changes, oversensing, or arrhythmias.

The SyncAV algorithm (Abbott) extends the paced AV delay every 256 cycles to allow 3 intrinsic beats, measuring intrinsic AV interval on the third beat.^1^^,^^2^ Subtracting the programmed offset (default 50 ms) recalibrates the AV delay for the next 256 cycles.^1^ During these 3 beats, the extended AV delay permits greater intrinsic conduction, producing a transient change in fusion morphology, a self-terminating, 3-beat pattern characteristic of normal SyncAV function.

Other manufacturers use dynamic CRT algorithms that similarly alter surface ECG patterns.

• Medtronic (AdaptivCRT): Periodically evaluates intrinsic AV conduction. If normal, it switches to synchronized LV-only pacing, but if prolonged, it provides adaptive biventricular pacing with optimized AV and ventriculo-ventricular (VV) intervals.
• Biotronik (CRT AutoAdapt): Occasionally assesses AV and VV intervals, switching to LV-only pacing when the intrinsic right ventricular conduction precedes LV conduction, producing transient fusion beats that may resemble native conduction on ECG.
• Boston Scientific (SmartDelay): Calculates optimal AV and VV delays based on intracardiac electrograms, requiring periodic manual reprograming.

Recognizing that automated resynchronization algorithms regularly alter AV delays, pacing modes, or fusion degrees prevents misinterpreting normal device behavior as loss of capture or malfunction.

Choice A is incorrect; LV noncapture creates a QS pattern in V_1_ with persistent pacing spikes and does not self-resolve in 3 beats.^3^ Choice B is incorrect; automatic mode switch causes a sustained nontracking mode transition (VVI/DDI/DVI) lasting seconds to minutes.^1^ Choice C is incorrect; right ventricular noncapture produces a right bundle branch block–like pattern in V_1_ and persistent threshold/impedance changes.^1^

## Funding Support and Author Disclosures

The authors have reported that they have no relationships relevant to the contents of this paper to disclose.
